# Overcoming Dilution of Collision Probability in Satellite Conjunction Analysis via Confidence Distribution

**DOI:** 10.3390/e27040329

**Published:** 2025-03-21

**Authors:** Hangbin Lee, Youngjo Lee

**Affiliations:** 1 Department of Information and Statistics, Chungnam National University, Daejeon 34134, Republic of Korea; hangbin221@gmail.com; 2 Department of Statistics, Seoul National University, Seoul 08826, Republic of Korea

**Keywords:** confidence distribution, epistemic confidence, probability dilution, satellite conjunction analysis

## Abstract

In satellite conjunction analysis, dilution of collision probability has been recognized as a significant deficiency of probabilistic inference. A recent study identified the false confidence problem as another limitation and suggested a possible causal link between the two, arguing that addressing false confidence could be necessary to prevent dilution of collision probability. However, this paper clarifies the distinction between probability dilution and false confidence by investigating a confidence distribution (CD) with a point mass at zero. Although the point mass in CD has often been perceived as paradoxical, we demonstrate that it plays an essential role in satellite conjunction analysis by capturing the uncertainty in the data. Consequently, the CD resolves the probability dilution of probabilistic inference and the ambiguity in Neymanian confidence intervals, while addressing the false confidence for the hypothesis of interest in satellite conjunction analysis. Furthermore, the confidence derived from the CD offers a direct interpretation as a *p*-value for hypothesis testing related to collision risk.

## 1. Introduction

In satellite conjunction analysis, accurately assessing collision probability (risk) is essential for ensuring the safety of satellite operations and maintaining the sustainability of the space environment. Underestimation or overestimation of risk exposure can distort assessments and result in suboptimal decisions. This may lead to unnecessary avoidance maneuvers that waste resources or, conversely, overlooked risks that result in collisions. Probability dilution [1], recognized as a fundamental deficiency of probabilistic inference, occurs when the assessed collision risks converge to zero as the uncertainty (noise) in the data increases [2]. This phenomenon could lead to a misinterpretation that lower-quality data appears to reduce the probability of collision. Therefore, effectively addressing probability dilution is crucial for enhancing decision-making processes and minimizing operational disruptions, especially with the growing density of satellite traffic.

Lee [3] demonstrated that the original Bayes-Laplace [4,5] solution for the so-called sunrise problem also suffers from probability dilution. A similar phenomenon was reported in the statistical literature [6], known as Stein’s length problem, where probabilistic inference for the Euclidean distance exhibits paradoxical behavior as the dimension of the data increases. Stein’s length problem suffers a severe probability dilution with an increasing number of parameters, whereas the satellite conjunction analysis suffers probability dilution with increasing variance. Pawitan and Lee [7] showed that the confidence is not a probability but a likelihood. This paper shows that the use of confidence distribution (CD) can overcome the probability dilution in satellite conjunction analysis, as well as in Stein’s length problem. The concept of CD has been proposed to offer probabilistic inference within the context of frequentist coverage probability. Although the fiducial distribution (FD; [8]) with a similar concept has faced heavy criticism, there has been a surge of renewed interest in CD with its modern definition over the past decade [9,10]. Lee and Lee [11] extended the confidence approach to the models with additional random unknowns.

In line with this concept, Cunen et al. [12] proposed the use of CD for satellite conjunction analysis as an alternative to the Bayesian approach to avoid probability dilution. However, their approach encountered strong criticism from Martin et al. [13], who argued that not only Bayesian posteriors but also the CD are susceptible to false confidence [2]. They further suggested that an additional consonant feature [14] is necessary for CD to address the false confidence [13,15,16]. Nevertheless, the concern over false confidence in satellite conjunction analysis could appear to be somewhat exaggerated, potentially imposing unnecessary restrictions on exploring alternative methods. Through an investigation of the CD with a point mass at zero, we demonstrate that addressing false confidence is not essential for preventing probability dilution. Instead, we highlight that the confidence feature is key to preventing probability dilution, which refers to the property where the assigned confidence of an observed interval exactly matches the actual coverage probability of the interval estimation procedure.

In Section 2, we present the background theory on probability dilution, false confidence, and CD. In Section 3, we identify a deficiency in the Neymanian confidence interval (CI) procedure for satellite conjunction analysis, where the confidence level of an observed CI can be ambiguous. In Section 4, we investigate the use of CD in satellite conjunction analysis and demonstrate that the CD resolves the ambiguity in Neymanian CI procedures. In Section 5, we investigate related methods in satellite conjunction analysis, including the generalized fiducial distribution (GFD; [17]) and the reference posterior (RP; [18]), for comparison with the CD. In Section 6, we highlight the advantages of the CD in overcoming probability dilution and providing a direct interpretation for hypothesis testing.

## 2. Backgrounds

### 2.1. Probability Dilution in Satellite Conjunction Analysis

The goal of satellite conjunction analysis is to assess the collision risk between two satellites and determine whether an avoidance maneuver is necessary or not. However, the estimated satellite trajectories have random measurement errors (noise), which can be represented by $\epsilon ∼N(0,\sigma^{2})$ with known $\sigma^{2}$, while the true satellite trajectories are fixed unknowns. Bayesian approaches appear to be widely used for assessing the collision probability, since it offers a probabilistic inference for true distance between the two satellites. However, a counterintuitive phenomenon known as the probability dilution has been recognized, where the collision probability tends to approach zero as the variance of random noise increases. Ad hoc methods have been proposed to address such a probability dilution, e.g., introducing alternative risk metrics [19,20,21] or adopting a high sensitivity to small collision probabilities [22], but Balch et al. [2] argued that it is not immediately obvious that being sensitive to small collision probabilities will resolve the problem.

Let $\mu_{a}=\left(\mu_{a1},\mu_{a2},\mu_{a3}\right)$ and $\mu_{b}=\left(\mu_{b1},\mu_{b2},\mu_{b3}\right)$ represent the 3-dimensional coordinates of the true locations of two satellites *A* and *B*, respectively. Then, let $x_{a}∼N\left(\mu_{a},\Sigma_{a}\right)$ and $x_{b}∼N\left(\mu_{b},\Sigma_{b}\right)$ represent their measurements of the satellites *A* and *B*, respectively, having multivariate normal distributions [1,2,12,13,23,24,25,26]. The difference of two measured locations has a multivariate normal distribution,

$$x_{a}-x_{b}∼N\left(\mu_{a}-\mu_{b},\;\Sigma \right),$$

where Σ denotes the covariance matrix, which can be decomposed by $\Sigma =Q\Lambda Q^{T}$ with an orthogonal matrix *Q* of the eigenvectors of Σ and a diagonal matrix Λ of the eigenvalues of Σ. Then, the principal axis alignment of the three-dimensional ellipsoid from the original coordinates x to the rotated coordinates $\tilde{x}=Q^{T}x$ simplifies the satellite conjunction analysis by removing off-diagonal correlation terms [2,12,13,25,26]. Thus, for notational convenience, the true Euclidean distance between the two satellites (parameter of interest) is denoted by

$$\theta =\sqrt{\theta_{1}^{2}+\theta_{2}^{2}+\theta_{3}^{2}},$$

where $\theta_{i}=\mu_{ai}-\mu_{bi}$ is the *i*th coordinate of the true difference between the two satellites, and the measurement of θ is denoted by

$$d=\sqrt{y_{1}^{2}+y_{2}^{2}+y_{3}^{2}},$$

where $y_{i}=x_{ai}-x_{bi}∼N\left(\theta_{i},\sigma_{a}^{2}+\sigma_{b}^{2}\right)$ for i=1,2,3, and $\sigma_{a}^{2}$ and $\sigma_{b}^{2}$ are noise levels of satellites A and B, respectively. The parameter space Θ and sample space $\Omega_{D}$ are identical to [0,∞). For clarity of notation throughout the paper, random variables are denoted by uppercase letters, such as $Y_{1}$, $Y_{2}$, $Y_{3}$, and *D*, while their observed values are denoted by lowercase letters, such as $y_{1}$, $y_{2}$, $y_{3}$, and *d*.

The collision of two spherical objects in 3D space is often simplified to the collision of two disks in a 2D impact plane [2,12,13,25,26], where the measurements of true distance between the two satellites in the two-dimensional plane are $Y_{i}∼N\left(\theta_{i},\sigma^{2}\right)$ for i=1,2. For ease of understanding, examples and numerical studies follow this simplified two-dimensional structure, while the theoretical results are derived in the general three-dimensional structure.

In Stein’s length problem, $Y_{1},...,Y_{k}$ are assumed to be independent random variables with $Y_{i}∼N\left(\theta_{i},\sigma^{2}\right)$ for i=1,...,k. The parameter of interest is

$$\theta =\sqrt{\sum_{i=1}^{k}\theta_{i}^{2}}.$$

When θ=0, the statistic $\sum_{i=1}^{k}Y_{i}^{2}$ follows a central Chi-squared distribution, and when θ>0, it follows a non-central Chi-squared distribution. Stein [6] demonstrated probability dilution in the marginal posterior under the flat prior when θ≪k. Results of this paper can also be applied to Stein’s length problem; however, we primarily focus on the satellite problem, reducing it to k=2 for simplicity in our discussions.

### 2.2. Confidence Distribution

The frequentist approach strictly distinguishes between the uncertainty of fixed unknowns (true trajectories) and the uncertainty of random unknowns (measured trajectories). In this view, the uncertainty of fixed unknowns can be represented by the confidence levels of a CI procedure. However, we point out that the Neymanian CI procedure becomes ambiguous in satellite conjunction analysis, as multiple CI procedures with different confidence levels can produce the same observed CI. Fisher [8] proposed the concept of FD as an alternative to the Bayesian posterior without assuming a prior, but it has faced significant controversies, such as its non-coherency [27] and adherence to Kolmogorov axioms [28]. The concept of CD has been developed with a modern definition to avoid widespread controversies of FD.

**Definition** **1** (CD [9]). *A non-decreasing right-continuous function C(θ;y) of the one-dimensional parameter θ and the data Y=y is the cumulative distribution function for a CD for θ, provided it has a uniform distribution under $P_{\theta ,\psi }$, whatever the true value of θ and nuisance parameters $\psi ={\left(\psi_{1},...,\psi_{p}\right)}^{\prime }$*.

Suppose that $CI_{\alpha }\left(Y\right)$ denotes the α-level CI procedure for the parameter of interest θ and $CI_{\alpha }\left(y\right)$ denotes an observed CI with the data Y=y. In this paper, the confidence feature of CD indicates that

(1) $$C\left(\theta \in CI_{\alpha }\left(y\right)\right)=\alpha =P_{\theta }\left(\theta \in CI_{\alpha }\left(Y\right)\right),$$

where $C(\theta \in CI_{\alpha }\left(y\right))$ is the epistemic confidence of an observed CI from the CD and $P_{\theta }\left(\theta \in CI_{\alpha }\left(Y\right)\right)$ is the aleatory coverage probability of the CI procedure. In other words, the confidence feature ensures that the assigned confidence of an observed interval aligns with its coverage probability. It is worth noting that the use of CD requires care in multi-parameter cases. While integrating out nuisance parameters is legitimate to obtain the marginal posterior of the parameter of interest in Bayesian analysis, integrating CD may no longer result in a valid CD, analogous to the marginalization paradox [29]. Stein’s length problem serves as a benchmark for illustrating this phenomenon of the integrated CDs, which leads to the CIs exhibiting undesirable behavior [9,27]. Pawitan and Lee [7] showed that the CD is not a probability but an extended likelihood, which is necessary to avoid probability-related paradoxes [30]. Clearly, integrating out nuisance parameters is not a legitimate way to obtain the marginal extended likelihood. The extended likelihood principle [31] states that the extended likelihood contains all the information in the data. Pawitan et al. [32] proposed the CD associated with the full data likelihood, which leaves no room for a relevant subset. They obtained a marginal CD with the full data likelihood by conditioning on maximal ancillary statistics.

## 3. Ambiguity in Confidence Level of an Observed CI

We first demonstrate that an observed CI can exhibit ambiguity in its confidence level (coverage probability). Suppose that we construct a CI procedure based on the statistics *D*, which is a measurement of the true distance θ in three-dimensional space, having

(2) $$\frac{D^{2}}{\sigma_{a}^{2}+\sigma_{b}^{2}}∼\chi_{3}^{2}\left(\frac{\theta^{2}}{\sigma_{a}^{2}+\sigma_{b}^{2}}\right),$$

where $\chi_{df}^{2}\left(\nu \right)$ denotes the non-central Chi-square distribution with the degrees of freedom df and non-centrality parameter ν. Suppose that $q_{\alpha }\left(\theta \right)$ is the (1−α) quantile function of *D* such that

(3) $$P_{\theta }\left(D\leq q_{\alpha }\left(\theta \right)\right)=1-\alpha ,$$

where α∈(0,1). Since $q_{\alpha }\left(\theta \right)$ is a strictly increasing function of θ for any α∈(0,1), there exists an inverse function $q_{\alpha }^{-1}\left(d\right)$ such that

(4) $$P_{\theta =q_{\alpha }^{-1}\left(d\right)}\left(D\leq d\right)=P_{\theta }\left(q_{\alpha }^{-1}\left(D\right)\leq \theta \right)=1-\alpha .$$

However, the range of $q_{\alpha }\left(\theta \right)$ is $\left[q_{\alpha }\left(0\right),\infty \right)$, where $q_{\alpha }\left(0\right)$ is the (1−α) quantile of the central Chi-square distribution. As $q_{\alpha }^{-1}\left(d\right)$ is not defined for $d<q_{\alpha }\left(0\right)$, we let $q_{\alpha }^{-1}\left(d\right)=0$ for such *d*. For α=0 and 1, let $q_{0}^{-1}\left(d\right)=\lim_{\alpha \to 0}q_{\alpha }^{-1}\left(d\right)=0$ and $q_{1}^{-1}\left(d\right)=\lim_{\alpha \to 1}q_{\alpha }^{-1}\left(d\right)=\infty$.

We investigate the following two Neymanian CI procedures with the same confidence level α and different endpoints,

(5) $${\mathrm{CI}}_{\alpha }\left(D\right)=\left[\theta_{L}\left(D\right),\theta_{U}\left(D\right)\right),$$

and

(6) $${\mathrm{CI}}_{\alpha }^{*}\left(D\right)=\left[\theta_{L}\left(D\right),\theta_{U}\left(D\right)\right],$$

where $\theta_{L}\left(D\right)=q_{1-\alpha -\beta }^{-1}\left(D\right)$ and $\theta_{U}\left(D\right)=q_{1-\beta }^{-1}\left(D\right)$ for some 0≤β≤1−α. ${\mathrm{CI}}_{\alpha }^{*}\left(D\right)$ is a closed interval, whereas ${\mathrm{CI}}_{\alpha }\left(D\right)$ is a half-closed interval. When β=0 or 1−α, the CI procedure become one-sided, either $\left[\theta_{L}\left(D\right),\infty \right)$ or $\left[0,\theta_{U}\left(D\right)\right)$, respectively. When 0<β<1−α, it becomes two-sided. For example, if α=0.9 and β=0.05, then $\theta_{L}\left(D\right)=q_{0.05}^{-1}\left(D\right)$ and $\theta_{U}\left(D\right)=q_{0.95}^{-1}\left(D\right))$ with the confidence level α=0.95−0.05=0.9. The coverage probabilities of the CI procedures (5) and (6) are equivalent to the confidence level α for all θ>0:

$$\begin{array}{cc} P_{\theta }\left(\theta \in {\mathrm{CI}}_{\alpha }\left(D\right)\right)\; & =P_{\theta }\left(\theta_{L}\left(D\right)\leq \theta <\theta_{U}\left(D\right)\right)\\ \; & =P_{\theta }\left(\theta_{L}\left(D\right)\leq \theta \right)-P_{\theta }\left(\theta_{U}\left(D\right)\leq \theta \right)=\alpha +\beta -\beta =\alpha ,\\ P_{\theta }\left(\theta \in {\mathrm{CI}}_{\alpha }^{*}\left(D\right)\right)\; & =P_{\theta }\left(\theta_{L}\left(D\right)\leq \theta \leq \theta_{U}\left(D\right)\right)\\ \; & =P_{\theta }\left(\theta_{L}\left(D\right)\leq \theta \right)-P_{\theta }\left(\theta_{U}\left(D\right)<\theta \right)=\alpha +P_{\theta }\left(\theta_{U}\left(D\right)=\theta \right)=\alpha . \end{array}$$

However, for θ=0, the CI procedure (5) has

$$P_{\theta =0}\left(0\in {\mathrm{CI}}_{\alpha }\left(D\right)\right)=P_{\theta =0}\left(\theta_{L}\left(D\right)=0<\theta_{U}\left(D\right)\right)=\alpha ,$$

whereas the CI procedure (6) has

$$P_{\theta =0}\left(0\in {\mathrm{CI}}_{\alpha }^{*}\left(D\right)\right)=P_{\theta =0}\left(\theta_{L}\left(D\right)=0\leq \theta_{U}\left(D\right)\right)=\alpha +P_{\theta =0}\left(\theta_{L}\left(D\right)=\theta_{U}\left(D\right)=0\right)>\alpha .$$

For an observed data D=d, the two-sided CI procedure (5) with 0<β<1−α leads to the following observed interval, ${\mathrm{CI}}_{\alpha }\left(d\right)=\left[\theta_{L}\left(d\right),\theta_{U}\left(d\right)\right)$:

- If $d>q_{1-\alpha -\beta }\left(0\right)$, the observed CI becomes two-sided interval $\left[\theta_{L}\left(d\right),\theta_{U}\left(d\right)\right)$ with $\theta_{L}\left(d\right)>0$.
- If $q_{1-\beta }\left(0\right)<d\leq q_{1-\alpha -\beta }\left(0\right)$, the observed CI becomes one-sided interval $\left[0,\theta_{U}\left(d\right)\right)$.
- If $d\leq q_{1-\beta }\left(0\right)$, the observed CI becomes an empty interval [0,0).

On the other hand, the CI procedure (6) leads to the following observed CI:

- If $d>q_{1-\alpha -\beta }\left(0\right)$, the observed CI becomes two-sided closed interval $\left[\theta_{L}\left(d\right),\theta_{U}\left(d\right)\right]$.
- If $q_{1-\beta }\left(0\right)<d\leq q_{1-\alpha -\beta }\left(0\right)$, the observed CI becomes one-sided closed interval $\left[0,\theta_{U}\left(d\right)\right]$.
- If $d\leq q_{1-\beta }\left(0\right)$, the observed CI becomes {0}.

The main difference between the observed CIs from (5) and (6) occurs in case c: (5) provides an empty interval and (6) provides an interval {0}. Although the empty CI seems not natural, it is important to maintain the confidence feature that only the procedure (5) maintains the coverage probability α for all θ∈Θ, including θ=0, whereas the procedure (6) gives a conservative interval at θ=0. However, the open CI procedure $\left(\theta_{L}\left(D\right),\theta_{U}\left(D\right)\right)$ cannot maintain the coverage probability, as we shall show.

**Figure 1:**
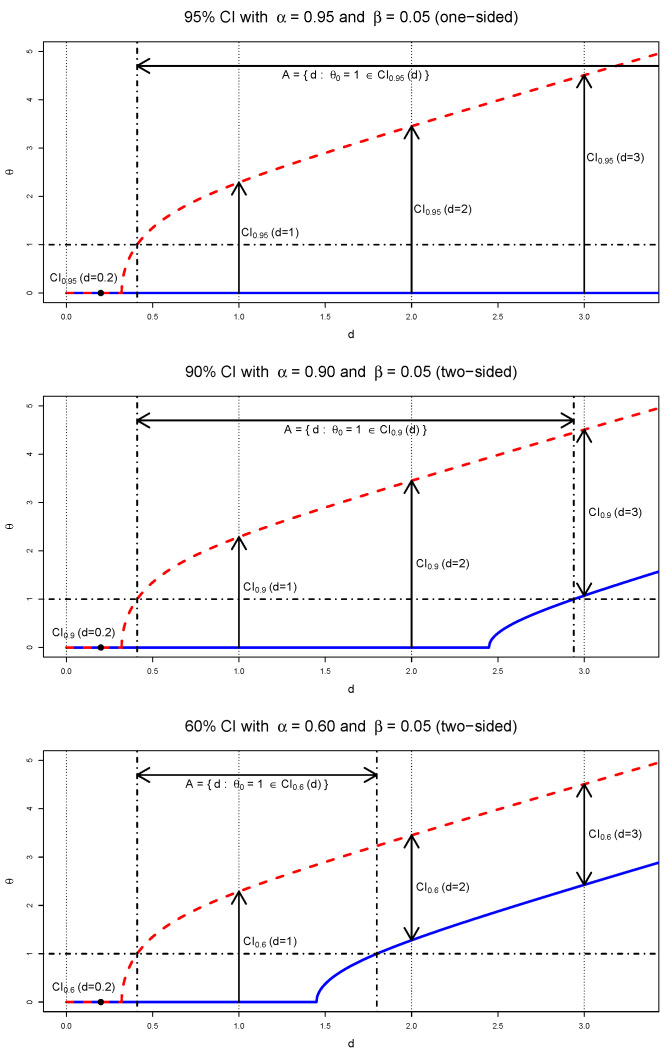
Upper bounds (red) and lower bounds (blue) of the confidence intervals with α = 0.95, 0.90, and 0.60 (from top to bottom).

As an illustrative example, consider the case where σ=1 and β=0.05. Figure 1 presents three CI procedures (5) with different confidence level; one-sided CI procedure with $\theta_{L}\left(D\right)=0$ for α=0.95 and two-sided CI procedures for α=0.90 and 0.60. All three CIs have a common upper bound, $\theta_{U}\left(d\right)=q_{1-\beta }^{-1}\left(d\right)=q_{0.95}^{-1}\left(d\right)$. In the figures, the horizontal axis and vertical axis represent *d* and θ, respectively. Dashed lines and the solid lines represent $\theta_{U}\left(d\right)$ and $\theta_{L}\left(d\right)$, respectively. For α=0.90 and 0.60, the two-sided CI procedure yields a two-sided observed CI when d>2.448 and d>1.449, respectively. When d≤0.320, all three CI procedures result in empty intervals. In Figure 1, horizontal arrows indicate the area $A=\{\;d:\theta_{0}=1\in {\mathrm{CI}}_{\alpha }\left(d\right)\;\}$, where $\theta_{0}$ is the true value of θ. Thus, if d∈A, the observed CI contains the true parameter value $\theta_{0}=1$. Furthermore, $P\left(A\right)=P(\theta_{0}\in {\mathrm{CI}}_{\alpha }\left(D\right))=\alpha$ implies that these three Neymanian CI procedures have the correct coverage probabilities. Vertical arrows indicate the observed CIs at d=1, 2, and 3. For instance, if d=2, the observed CI [0,3.451) could be a realization of either a 95% or 90% Neymanian CI procedure, i.e.,

$${\mathrm{CI}}_{0.95}\left(d=2\right)={\mathrm{CI}}_{0.9}\left(d=2\right)=\left[0,3.451\right).$$

Similarly, if d=1, the observed CI [0,2.287) could be a realization of a 60%, 90% or 95% Neymanian CI procedure, i.e.,

$${\mathrm{CI}}_{0.95}\left(d=1\right)={\mathrm{CI}}_{0.9}\left(d=1\right)={\mathrm{CI}}_{0.6}\left(d=1\right)=\left[0,2.287\right).$$

Consequently, given an observed CI, its actual coverage probability (confidence level) may not be uniquely determined. This raises questions about the meaning of confidence level for an observed CI.

## 4. CD for Satellite Conjunction Analysis

With the two-dimensional setting for the satellite conjunction analysis, let (θ,ψ) and (D,T) be the polar coordinate representations of $\left(\theta_{1},\theta_{2}\right)$ and $\left(Y_{1},Y_{2}\right)$, respectively:

$$\left(\theta_{1},\theta_{2}\right)=\left(\theta \cos\psi ,\theta \sin\psi \right)\;\mathrm{and}\;\left(Y_{1},Y_{2}\right)=\left(D\cos T,D\sin T\right),$$

where $\psi =\tan^{-1}\left(\theta_{2}/\theta_{1}\right)$ and $T=\tan^{-1}\left(Y_{2}/Y_{1}\right)$. Here, the distributions of *T* and T|D still depend on both θ and ψ, hence *D* alone is not a sufficient statistic for θ under the full data $\left(y_{1},y_{2}\right)$. Pawitan et al. [32] proposed the use of conditional distribution D|A for a maximal ancillary statistic *A* to derive a CD with full data information. However, maximal ancillary statistic is not known in satellite conjunction analysis. In such cases, the CD is marginally defined for the parameter of interest [27], though there could be a loss of information. The current definition of the CD only requires that

(7) $$C\left(\theta_{0};D\right)∼\mathrm{Uniform}\left[0,1\right],$$

where $C(\theta_{0};D)$ denotes the CD at the true value $\theta_{0}$ of θ, which guarantees that the CD maintains the confidence feature [9]. Cunen et al. [12] derived the marginal CD for θ, based on the observed statistic D=d,

(8) $$C\left(\theta ;d\right)=P_{\theta }\left(D\geq d\right)=1-\Gamma_{2}\left(\frac{d^{2}}{\sigma^{2}};\frac{\theta^{2}}{\sigma^{2}}\right),$$

where $\Gamma_{2}\left(\;\cdot \;;\nu \right)$ denotes the cumulative distribution function of the non-central Chi-square distribution with degrees of freedom 2, and they showed that this CD does not have probability dilution. This coincides with Wilkinson’s [27] marginal CD for Stein’s problem with k>2.

With the three-dimensional setting for the satellite conjunction analysis, similarly we let $\left(\theta ,\psi_{1},\psi_{2}\right)$ and $\left(D,T_{1},T_{2}\right)$ be the spherical coordinate representations of $\left(\theta_{1},\theta_{2},\theta_{3}\right)$ and $\left(Y_{1},Y_{2},Y_{3}\right)$, respectively:

$$\begin{array}{cc} \left(\theta_{1},\theta_{2},\theta_{3}\right) & =(\theta \sin\psi_{1}\cos\psi_{2},\theta \sin\psi_{1}\sin\psi_{2},\theta \cos\psi_{1}),\\ \left(Y_{1},Y_{2},Y_{3}\right) & =(D\sin T_{1}\cos T_{2},D\sin T_{1}\sin T_{2},D\cos T_{1}), \end{array}$$

where

$$\begin{array}{cc} \psi_{1} & =\cos^{-1}\left(\frac{\theta_{3}}{\sqrt{\theta_{1}^{2}+\theta_{2}^{2}+\theta_{3}^{2}}}\right),\;\psi_{2}=\mathrm{sign}\left(\theta_{2}\right)\cos^{-1}\left(\frac{\theta_{1}}{\sqrt{\theta_{1}^{2}+\theta_{2}^{2}}}\right),\\ T_{1} & =\cos^{-1}\left(\frac{Y_{3}}{\sqrt{Y_{1}^{2}+Y_{2}^{2}+Y_{3}^{2}}}\right),\;T_{2}=\mathrm{sign}\left(Y_{2}\right)\cos^{-1}\left(\frac{Y_{1}}{\sqrt{Y_{1}^{2}+Y_{2}^{2}}}\right), \end{array}$$

then we can see that *D* is still not a sufficient statistic for θ under the full data $\left(y_{1},y_{2},y_{3}\right)$. The marginal CD for θ based on the observed statistic D=d becomes

(9) $$C\left(\theta ;d\right)=P_{\theta }\left(D\geq d\right)=1-\Gamma_{3}\left(\frac{d^{2}}{\sigma_{a}^{2}+\sigma_{b}^{2}};\frac{\theta^{2}}{\sigma_{a}^{2}+\sigma_{b}^{2}}\right),$$

where $\Gamma_{3}\left(\;\cdot \;;\nu \right)$ denotes the cumulative distribution function of non-central Chi-square distribution with degrees of freedom 3.

With a slight abuse of notation, we denote the confidence assigned to a proposition A⊂Θ as follows:

(10) $$C\left(A\right)=C\left(A;d\right)=C\left(\theta \in A\right)=\int_{A}c\left(\theta ;d\right)d\theta ,$$

where c(θ;d) is a confidence density. Then, the CD (8) for two-dimension leads to

(11) $$C\left(\theta_{0};D\right)=C\left(\left[0,\theta_{0}\right];D\right)=1-\Gamma_{2}\left(\frac{D^{2}}{\sigma^{2}};\frac{\theta_{0}^{2}}{\sigma^{2}}\right)∼\mathrm{Uniform}\left[0,1\right],$$

and the CD (8) for three-dimension leads to

(12) $$C\left(\theta_{0};D\right)=C\left(\left[0,\theta_{0}\right];D\right)=1-\Gamma_{3}\left(\frac{D^{2}}{\sigma_{a}^{2}+\sigma_{b}^{2}};\frac{\theta_{0}^{2}}{\sigma_{a}^{2}+\sigma_{b}^{2}}\right)∼\mathrm{Uniform}\left[0,1\right],$$

i.e., they give correct coverage probabilities for any true value $\theta_{0}\in \Theta =\left[0,\infty \right)$.

### 4.1. Point Mass in CD

This section shows that the presence of a point mass is not a drawback but an advantage, as the point mass plays an essential role in maintaining the coverage probability. At θ=0, the CD becomes

(13) $$C\left(\left\{0\right\}\right)=1-\Gamma_{2}\left(\frac{d^{2}}{\sigma^{2}};0\right)\geq 0.$$

Wilkinson [27] interpreted the point mass at θ=0 as an unassigned probability. He assumed the parameter space as Θ=(0,∞), so that the CD is not a probability distribution because C(Θ)<1. In this context, the point mass at zero looks paradoxical [9]. However, since $\theta_{1}=0$ and $\theta_{2}=0$ are originally included in the parameter space, we let Θ=[0,∞). Then, C(Θ)=1 holds without an unassigned probability.

Let M(D)=C({0};D) denote the point mass at θ=0 and M(d) denote the realized value of the point mass given an observation D=d. The confidence density c(θ;d) can be expressed as

(14) $$c\left(\theta ;d\right)=M\left(d\right)\;\cdot \;D\left(\theta \right)+c_{+}\left(\theta ;d\right),$$

where D(θ) denotes the Dirac delta function to give a point mass at θ=0 and $c_{+}\left(\theta ;d\right)=\partial P_{\theta }\left(D\geq d\right)/\partial \theta$ for θ>0. It is worth emphasizing that the point mass M(D) captures the uncertainty of data. If σ→0 or θ→∞, the point mass M(D) vanishes

$$M\left(D\right)\overset{p}{\to }1-\Gamma_{2}\left(\infty ;0\right)=0.$$

If σ→∞ or θ→0,

$$M\left(D\right)\overset{d}{\to }\mathrm{Uniform}\left[0,1\right].$$

This property ensures that the CD does not suffer from probability dilution and maintain the confidence feature even if $\sigma^{2}\to \infty$. Theorem 1 identifies a necessary and sufficient condition for the existence of a point mass in CD, where θ∈Θ is the parameter of continuous scalar statistic $D\in \Omega_{D}$ and the 1−α quantile $q_{\alpha }\left(\theta \right)$ is a strictly increasing function of θ for any α∈(0,1). The proof is in Appendix A.

**Theorem** **1.** *Let $\partial \Omega_{D}$ and ∂Θ denote the boundary of $\Omega_{D}$ and Θ, respectively. Then, C(θ;d) has no point mass if and only if*

(15) $$q_{\alpha }\left(\theta \right)\to \partial \Omega_{D}\;\mathrm{as}\;\theta \to \partial \Theta ,\;\mathrm{for}\;\mathrm{any}\;\alpha \in \left(0,1\right).$$

**Remark** **1.** *Pawitan et al. [32] considered a curved exponential model. Let y=1 be an observation from $Y∼N(\theta ,\theta^{2})$ for θ≥0, then one may consider a confidence distribution,*

$$C\left(\theta ;y\right)=P_{\theta }\left(Y\geq y\right)=1-\Phi \left(\frac{y-\theta }{\theta }\right),$$

*where Φ(·) denotes the cumulative function of N(0,1). However, this leads to*

$$\lim_{\theta \to \infty }C\left(\theta ;y\right)=1-\Phi \left(-1\right)\approx 0.84<1.$$

*Thus, C(θ;y) cannot be a cumulative distribution function of θ. Here C({0};y=1)=0, i.e., there is no point mass at θ=0. Following Wilkinson [27], one may say that this CD has an unassigned probability 0.16=1−0.84. This problem occurs because the quantile function $q_{\alpha }\left(\theta \right)$ is not increasing function of θ. Now let d=|y| be an observation of D=|Y| with $\Omega_{D}=\Theta$. Then the corresponding CD is defined as*

$$C\left(\theta ;d\right)=P_{\theta }\left(D\geq d\right)=1-\Phi \left(\frac{d-\theta }{\theta }\right)+\Phi \left(\frac{-d-\theta }{\theta }\right),$$

*which becomes a proper cumulative distribution function without a point mass*

$$\lim_{\theta \to 0}C\left(\theta ;d\right)=1-\Phi \left(\infty \right)+\Phi \left(-\infty \right)=0\;\mathrm{and}\;\lim_{\theta \to \infty }C\left(\theta ;d\right)=1-\Phi \left(-1\right)+\Phi \left(-1\right)=1.$$

*When there is no point mass, under appropriate conditions, Pawitan et al. [32] showed that*

(16) $$C\left(\theta \in CI\left(d\right)\right)=\int_{CI(d)}c\left(\theta ;d\right)d\theta =P_{\theta }\left(\theta \in CI\left(D\right)\right),$$

*where the LHS is the epistemic confidence of the observed CI and the RHS is the aleatory coverage probability of the CI procedure. This paper shows that confidence feature holds even with a point mass. In this paper, we use the term confidence for the observed CI, whereas the coverage probability (confidence level) for the CI procedure.*

**Remark** **2.** *In both Stein’s length problem and satellite conjunction analysis, lower bounds of $\Omega_{D}$ and Θ are assumed to be zero, whereas $q_{\alpha }\left(0\right)\neq 0\in \partial \Omega_{D}$. Thus, Theorem 1 implies that the corresponding CD has a point mass at zero.*

### 4.2. Confidence of an Observed CI

This section investigates the confidence of an observed CI from the CD, where the observed intervals ${\mathrm{CI}}_{\alpha }\left(d\right)$ correspond to those described in Section 3:

a. When the observed CI is two-sided with $\theta_{L}\left(d\right)>0$,
  $$C\left({\mathrm{CI}}_{\alpha }\left(d\right)\right)=C\left(\theta <\theta_{U}\left(d\right);d\right)-C\left(\theta <\theta_{L}\left(d\right);d\right)=\left(1-\beta \right)-\left(1-\alpha -\beta \right)=\alpha .$$
b. When the observed CI becomes one-sided with $\theta_{L}\left(d\right)=0$ and $\theta_{U}\left(d\right)>0$,
  $$C\left({\mathrm{CI}}_{\alpha }\left(d\right)\right)=C\left(\theta <\theta_{U}\left(d\right);d\right)=1-\beta =\max\left\{\;\alpha :{\mathrm{CI}}_{\alpha }\left(d\right)=\left[0,\theta_{U}\left(d\right)\right)\;\right\},$$
  which is the maximum confidence level (coverage probability) of the CI procedures having the same observed CI. In Figure 1, the CI procedures with α=0.95 and α=0.9 produce the same observed CI for d=2,
  $$\mathrm{CI}\left(d=2\right)={\mathrm{CI}}_{0.95}\left(d=2\right)={\mathrm{CI}}_{0.9}\left(d=2\right)=\left[0,3.451\right).$$
  For this observed CI, the CD gives confidence
  $$C\left(\left[0,3.451\right);d=2\right)=\max\left\{\;\alpha :{\mathrm{CI}}_{\alpha }\left(d=2\right)=\left[0,3.451\right)\;\right\}=0.95.$$
c. When the observed CI becomes an empty set with $\theta_{L}\left(d\right)=\theta_{U}\left(d\right)=0$, the point mass of the CD becomes
  $$M\left(d\right)=C\left(\left\{0\right\};d\right)=\max\left\{\;\alpha :{\mathrm{CI}}_{\alpha }\left(d\right)=\emptyset \;\right\},$$
  which is the maximum confidence level of the CI procedures having an empty observed CI. In Figure 1, all the three CI procedures produce an empty observed CI for d=0.2,
  $$\mathrm{CI}\left(d=0.2\right)={\mathrm{CI}}_{0.95}\left(d=0.2\right)={\mathrm{CI}}_{0.9}\left(d=0.2\right)={\mathrm{CI}}_{0.6}\left(d=0.2\right)=\emptyset .$$
  Here, the point mass of the CD is
  $$M\left(d=0.2\right)=C\left(\left\{0\right\};d=0.2\right)=\max\left\{\;\alpha :{\mathrm{CI}}_{\alpha }\left(d=0.2\right)=\emptyset \;\right\}=0.98,$$
  which implies that the CI procedure (5) produces an empty observed CI for α≤0.98.

The CD gives the maximum attainable confidence level (coverage probability) for a nonempty observed CI(d) from CI procedures (5),

$$C\left(\theta \in \mathrm{CI}\left(d\right)\right)=\max\;\left\{\;\alpha :{\mathrm{CI}}_{\alpha }\left(d\right)=\mathrm{CI}\left(d\right)\;\right\},$$

Furthermore, the CD gives the maximum attainable coverage probability for an observed ${\mathrm{CI}}^{*}\left(d\right)$ from CI procedure (6),

$$C\left(\theta \in {\mathrm{CI}}^{*}\left(d\right)\right)=\max\;\left\{\;\alpha :{\mathrm{CI}}_{\alpha }^{*}\left(d\right)={\mathrm{CI}}^{*}\left(d\right)\;\right\}.$$

Note here that CI(d)=∅ from (5) corresponds to ${\mathrm{CI}}^{*}\left(d\right)=\left\{0\right\}$ from (6). When d=0.2, all three intervals produce an observed interval,

$${\mathrm{CI}}^{*}\left(d=0.2\right)={\mathrm{CI}}_{0.95}^{*}\left(d=0.2\right)={\mathrm{CI}}_{0.9}^{*}\left(d=0.2\right)={\mathrm{CI}}_{0.6}^{*}\left(d=0.2\right)=\left\{0\right\}.$$

Here,

$$C\left(\left\{0\right\};d=0.2\right)=\max\left\{\;\alpha :{\mathrm{CI}}_{\alpha }^{*}\left(d=0.2\right)=\left\{0\right\}\;\right\}=0.98.$$

Previously, we showed the ambiguity in the confidence level of an observed CI. Given an observed CI, we see that its confidence is the maximum attainable confidence level of the possible CI procedures. Thus, when there exists a point mass in CD, it would be beneficial to report the confidence of the observed CI and the corresponding CI procedure.

## 5. Comparison with Related Methods

### 5.1. FD and GFD

Fisher [8] used a sufficient statistic to construct the FD. Since $\left(y_{1},y_{2}\right)$ are sufficient statistics for $\left(\theta_{1},\theta_{2}\right)$, consider the following joint cumulative function $G(\theta_{1},\theta_{2};y_{1},y_{2})$ as the FD for $\left(\theta_{1},\theta_{2}\right)$:

(17) $$G\left(\theta_{1},\theta_{2};y_{1},y_{2}\right)=P_{\theta_{1},\theta_{2}}\left(Y_{1}\geq y_{1}\;\mathrm{and}\;Y_{2}\geq y_{2}\right).$$

Then, corresponding density function is given by

$$g\left(\theta_{1},\theta_{2};y_{1},y_{2}\right)=\frac{\partial^{2}G\left(\theta_{1},\theta_{2};y_{1},y_{2}\right)}{\partial \theta_{1}\partial \theta_{2}}=\frac{1}{\sigma^{2}}\phi \left(\frac{\theta_{1}-y_{1}}{\sigma }\right)\phi \left(\frac{\theta_{2}-y_{2}}{\sigma }\right)=L\left(\theta_{1},\theta_{2};y_{1},y_{2}\right),$$

where ϕ(·) is the density function of N(0,1) and $L(\theta_{1},\theta_{2};y_{1},y_{2})$ is the likelihood function. Since it is identical to the joint posterior $\pi (\theta_{1},\theta_{2}|y_{1},y_{2})$ under $\pi (\theta_{1},\theta_{2})=1$, integration of the FD with respect to θ becomes identical to the marginal posterior $\pi (\theta |y_{1},y_{2})$,

(18) $$G\left(\theta ;d\right)=\int_{\theta_{1}^{2}+\theta_{2}^{2}\leq \theta^{2}}\frac{1}{\sigma^{2}}\phi \left(\frac{\theta_{1}-y_{1}}{\sigma }\right)\phi \left(\frac{\theta_{2}-y_{2}}{\sigma }\right)\;d\left(\theta_{1},\theta_{2}\right)=\Gamma_{2}\left(\frac{\theta^{2}}{\sigma^{2}};\frac{d^{2}}{\sigma^{2}}\right),$$

which gives the density,

(19) $$g\left(\theta ;d\right)=\frac{\partial G(\theta ;d)}{\partial \theta }=\frac{2\theta }{\sigma^{2}}\;\gamma_{2}\left(\frac{\theta^{2}}{\sigma^{2}};\frac{d^{2}}{\sigma^{2}}\right),$$

where $\gamma_{2}\left(\theta ;\;\cdot \;\right)=d\Gamma_{2}\left(\theta ;\;\cdot \;\right)/d\theta$. It has been shown that this integrated FD cannot maintain the correct coverage probability as k→∞ in the Stein’s length problem [6,27,33], and the same issue arises in the satellite conjunction analysis with k=2 as $\sigma^{2}\to \infty$.

**Figure 2:**
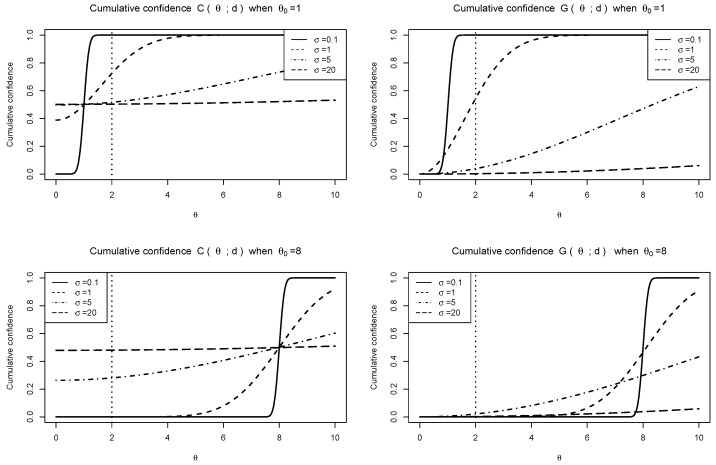
Average of C(θ;d) and G(θ,d) over 10,000 repeats.

Figure 2 shows the averages of cumulative functions from $10^{4}$ repetitions, based on C(θ;d) in (8) and G(θ;d) in (18), where the true $\theta_{0}$ is 1 or 8 and σ varies in {0.1,1,5,20}. Compared with the marginal CD C(θ;d), the integrated FD G(θ;d) exhibits apparent probability dilution: they become identical when σ→0 but differ significantly when σ becomes large. The figure also illustrates the point mass at zero, where C({0})≥0 but G({0})=0. Thus, while the use of G(θ;d) allows for two-sided observed CIs, it loses the confidence feature and suffers from probability dilution.

Generalized FD (GFD; [17]) is an extension of the FD to more general cases. In satellite conjunction analysis, the integrated FD can be viewed as a typical example of GFDs. Consider a data generating mechanism,

$$\left(Y_{1},Y_{2}\right)=\left(\theta \cos\theta +\sigma U_{1},\theta \sin\theta +\sigma U_{2}\right),$$

where $U_{1}$ and $U_{2}$ are independent random variables from N(0,1). The corresponding set-valued function is defined as

$$\begin{array}{cc} Q(y_{1},y_{2},U_{1}^{*},U_{2}^{*})\; & =\left\{\;\theta :\left(y_{1},y_{2}\right)=\left(\theta \cos\theta +\sigma U_{1}^{*},\theta \sin\theta +\sigma U_{2}^{*}\right)\;\right\}\\ \; & =\sigma \sqrt{{\left(\frac{y_{1}}{\sigma }-U_{1}^{*}\right)}^{2}+{\left(\frac{y_{2}}{\sigma }-U_{2}^{*}\right)}^{2}}∼\sigma \sqrt{\chi_{2}^{2}\left(\frac{d^{2}}{\sigma^{2}}\right)}, \end{array}$$

hence G(θ;d) satisfies the definition of GFD. It is worth noting that the marginal CD and Bayesian posteriors in the next section can also be included in the class of GFDs. However, since these integrated FD and Bayesian posteriors fail to maintain the correct coverage probability, GFD may not be a desirable generalization of CD with the confidence feature.

### 5.2. Bayesian Posteriors

In this section, abbreviations RP and UP denote the corresponding posteriors under the reference prior [18] and uniform (flat) prior, respectively. As demonstrated in Section 5.1, UP is identical to the integrated FD, which suffers from probability dilution. To resolve the probability dilution of UP in Stein’s length problem, Bernardo [18] proposed the use of RP, which can improve the UP with moderate *k* and $\sigma^{2}$. However, the RP cannot avoid the probability dilution entirely.

**Figure 3:**
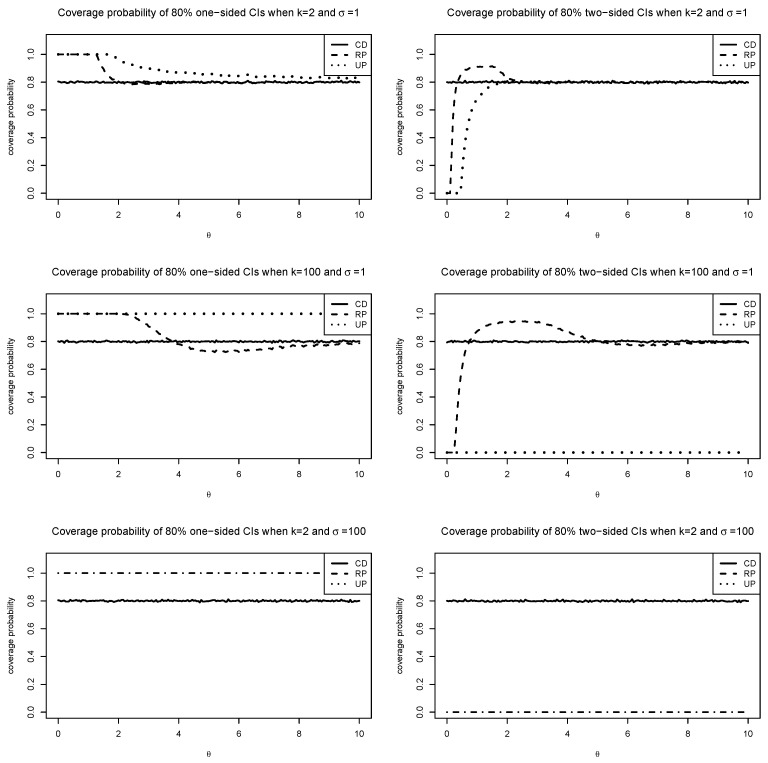
Coverage probabilities of 80% CIs based on CD, UP, and RP when k=2 and k=100.

Figure 3 shows the coverage probabilities of the one-sided and two-sided 80% CIs for satellite conjunction analysis (k=2) and Stein’s length problem (k=100), computed from $10^{4}$ repetitions. Probability dilution of UP is evident, especially when θ≪k. RP improves UP, but it also has probability dilution when θ is small or $\sigma^{2}$ is large. Note that the CD-based two-sided CI procedure automatically produces the one-sided observed CI to maintain the confidence feature when the observation *d* is small. On the other hand, since neither RP nor UP has a point mass at zero, they always allow for two-sided observed CIs, which implies that they cannot maintain the confidence feature. The figure shows that only the CD maintains the confidence feature for all θ∈Θ=[0,∞).

### 5.3. Consonant Belief

**Figure 4:**
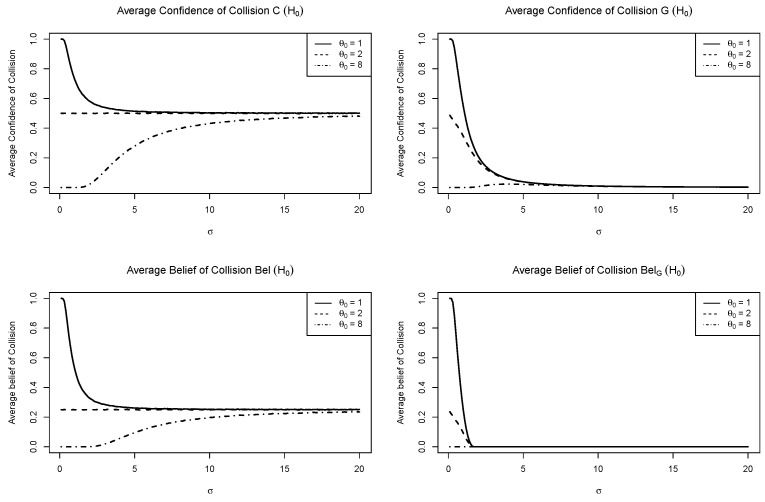
Average confidences and beliefs of collision over 10,000 repetitions.

Balch et al. [2] claimed the necessity of consonant feature [14] and proposed the use of consonant belief (CB) to prevent probability dilution,

(20) $$\mathrm{Bel}\left(A;d\right)=1-\underset{\theta \in A^{c}}{\sup}\mathrm{pls}\left(\theta ;d\right),$$

where Bel(·;d) is the consonant belief function and pls(θ;d)=1−|2C(θ;d)−1| is the plausibility contour. However, Figure 4 shows that the key to overcoming probability dilution is not the consonant feature but the confidence feature. The figure illustrates the average confidences and beliefs of collision as σ varies from 0 to 20. Suppose that $H_{0}:\theta \leq R$ is an assertion of collision. As σ increases, $C(H_{0})$ decreases to 0.5 when $\theta_{0}=1$, and $C(H_{0})$ increases to 0.5 when $\theta_{0}=8$. We see that these phenomena are caused by a point mass at zero: C({0})>0. $\mathrm{Bel}(H_{0})$ and ${\mathrm{Bel}}_{G}\left(\;\cdot \;\right)$ are CBs (20) based on CD and UP, respectively. $\mathrm{Bel}(H_{0})$ seems to prevent severe probability dilution as it converges to 0.223 as σ→∞. However, $\mathrm{Bel}\left(H_{0}\right)<C\left(H_{0}\right)$, which implies that the additional consonant feature reduces the confidence. On the other hand, as the UP has no point mass, $G(H_{0})$ goes to zero as σ→∞. Then, ${\mathrm{Bel}}_{G}\left(\left[0,\theta \right]\right)$ exhibits even more severe probability dilution. This experiment suggests that consonant feature may not prevent probability dilution.

The belief function [34,35] could be useful for trinary decision problems when combined with an additional plausibility function, as they serve as lower and upper bounds, respectively. However, Appendix B demonstrates that the belief function alone would not be suitable for making a binary decision rule, such as deciding whether to perform an avoidance maneuver or not in satellite conjunction analysis.

## 6. Further Advantages of CD

### 6.1. Direct Interpretation of CD for Hypothesis Testing

Hypothesis testing procedures are widely used in risk assessment for satellite conjunction analysis. As the null hypothesis $H_{0}$ is either θ≤R (collision) or θ>R (non-collision), where *R* is the sum of radii of the two satellites, collision probabilities are most commonly used as the test statistic [36]. For illustration, suppose that $H_{0}$ is the assertion of collision, so that the confidence-based collision risk can be directly evaluated by

(21) $$\mathrm{collision}\;\mathrm{risk}=C\left(H_{0};d\right)=C\left(R;d\right).$$

Then, from the property of CD,

(22) $$C\left(\theta \leq \theta_{0};D\right)∼\mathrm{Uniform}\left[0,1\right],$$

the confidence $C(H_{0};d)$ is directly interpreted as the observed *p*-value for testing $H_{0}$, i.e.,

(23) $$\max_{\theta \in H_{0}}P_{\theta }\left(C(H_{0};D)\leq \alpha \right)=\alpha .$$

Thus, the CD provides an α-level hypothesis testing procedure for any $\sigma^{2}$. However, since UP and RP do not have the point mass at zero,

(24) $$G\left(H_{0};D\right)\to 0\;\mathrm{and}\;R\left(H_{0};D\right)\to 0,$$

as $\sigma^{2}\to \infty$. Thus, if the data are of a poor quality with large $\sigma^{2}$, UP and RP fail to accept the null hypothesis even if d<R. For example, suppose that we observe d=1<R=2. When $\sigma^{2}=1$, the CD, UP and RP give $C(H_{0})=0.918$, $G(H_{0})=0.731$ and $R(H_{0})=0.891$, respectively. Thus, all of them would not reject $H_{0}$. Here, the RP becomes close to the CD. However, in satellite conjunction analysis, $\sigma^{2}$ is often much greater than *R* [2]. When σ=100, the CD yields $C(H_{0})=1.0$, hence the CD would not reject $H_{0}$. However, $G(H_{0})=0.000$ and $R(H_{0})=0.016<0.05$ to reject $H_{0}$ though the observed value implies the collision (d<R). Therefore, if the collision risk is given by the CD, there is no reason for engineers to ignore an impending collision risk due to the negligible confidence of collision. However, if it is based on either UP or RP, engineers may underestimate the impending danger because of the dilution of collision probability.

In Stein’s length problem it is often of interest to test

$$H_{0}:\;\theta =0\;\mathrm{vs}.\;H_{1}:\;\theta \neq 0.$$

Due to the point mass at θ=0, the CD gives

(25) $$P_{\theta \in H_{0}}\left(C\left(H_{0};D\right)<\alpha \right)=P_{\theta =0}\left(C\left(\left\{0\right\};D\right)<\alpha \right)=\alpha .$$

Thus, if we use $C(H_{0})$ as a *p*-value, we can directly achieve a valid hypothesis testing procedure with

(26) $$P_{H_{0}}\left(\mathrm{Reject}\;H_{0}\right)=P_{\theta =0}\left(C\left(H_{0}\right)<\alpha \right)=P_{\theta =0}\left(M\left(D\right)<\alpha \right)=\alpha .$$

On the other hand, the UP and RP have no point mass. Thus, $G(H_{0})$ and $R(H_{0})$ do not lead to a valid hypothesis testing, because $G\left(H_{0}\right)=R\left(H_{0}\right)=0$ for any observation *d*.

When $\sigma^{2}=\infty$, the data $\left(y_{1},y_{2}\right)$ are meaningless as an estimate of $\left(\theta_{1},\theta_{2}\right)$. However, poor data cannot justify the small collision probability $G(H_{0})\approx 0$ under impending collision situations. The low collision probability ($G(H_{0})$ or $R(H_{0})$) does not mean that the two satellites are far apart; it is only a statement of the general unlikelihood of such an alignment if all one knows is that the two satellites happen to be in the same general area [37]. However, it is undesirable for engineers to ignore impending danger because they believe a negligible collision probability caused by poor data quality. Since

(27) $$C\left(H_{0}\right)=1-C\left(H_{1}\right)\overset{d}{\to }\mathrm{Uniform}\left[0,1\right]$$

as $\sigma^{2}\to \infty$, the CD always acknowledges a non-negligible confidence of collision even with poor data. In this respect, the CD is useful for lowering the impending risk in satellite conjunction analysis.

### 6.2. Satisfying Martin-Liu Validity Criterion for the Proposition of Interest

Balch et al. [2] noted that the probabilistic inference can suffer from severe false confidence for a proposition *A*: if for some unacceptably high p∈(0,1) and unacceptably high 1−α where α∈(0,1), there exists some putative value of θ such that

$$\theta \notin A\;\mathrm{and}\;P_{\theta }\left\{C\left(A;D\right)\geq 1-\alpha \right\}\geq p.$$

They claimed that this false confidence is a fundamental deficiency in probabilistic inference by introducing the false confidence theorem below, under the assumption that $\sup_{\theta }c\left(\theta ;d\right)<\infty$ almost everywhere in *D* for any true $\theta_{0}\in \Theta$.

**Theorem** **2** (False Confidence Theorem [2]). *For any $\theta_{0}$, any α∈(0,1) and any p∈(0,1), there exists a proposition A⊂Θ such that $\theta_{0}\notin A$ and*

(28) $$P_{\theta_{0}}\left\{C\left(A;D\right)\geq 1-\alpha \right\}\geq p.$$

Martin and Liu [38] introduced Martin-Liu validity criterion, which requires protection for any false proposition: A statistical method is free from false confidence if for any α∈[0,1] and any false proposition A⊂Θ such that $\theta_{0}\notin A$,

(29) $$P_{\theta_{0}}\left\{C\left(A;D\right)\geq 1-\alpha \right\}\leq \alpha .$$

Martin et al. [13] argued that the false confidence theorem applies not only to Bayesian posteriors but also to the CD in satellite conjunction analysis, suggesting that an additional consonant feature is necessary for the CD. However, as demonstrated in Section 5.3, the key to overcoming probability dilution lies not in the consonant feature but in the confidence feature. Furthermore, the false confidence theorem cannot be applied to the CD, as the presence of a point mass at zero provides a counterexample: for any false proposition A⊆{θ:θ≠0} when $\theta_{0}=0$,

(30) C(A;D)≤C(θ≠0;D)=1−M(D)∼Uniform[0,1],

which leads to

(31) $$P_{\theta_{0}=0}\left\{C\left(A;D\right)\geq 1-\alpha \right\}\leq P_{\theta_{0}=0}\left\{M\left(D\right)\leq \alpha \right\}=\alpha .$$

Consequently, the CD satisfies the Martin-Liu validity criterion at least for the proposition of interest in satellite conjunction analysis. Suppose that $H_{0}$ (collision; θ≤R) is a true proposition and $H_{1}$ (non-collision; θ>R) is a false proposition *A*, where *R* is the sum of the radii of two satellites. Then, the level of false confidence becomes

(32) $$P_{\theta_{0}}\left\{C\left(H_{1};D\right)\geq 1-\alpha \right\}=P_{\theta_{0}}\left\{C\left(H_{0};D\right)\leq \alpha \right\}\leq P_{\theta_{0}}\left\{C\left(\theta_{0};D\right)\leq \alpha \right\}=\alpha .$$

Hence, if $H_{0}$ is true, the level of false confidence cannot grow arbitrarily large, i.e., the CD does not allow high false confidence for a false proposition $H_{1}$. This satisfies the Martin-Liu validity criterion (29) for $H_{1}$, which is the false proposition of interest. This paper argues that requiring the Martin-Liu validity criterion to be satisfied for any false proposition is not necessary, as it is not the key to preventing probability dilution and imposes severe restrictions on exploring alternative methods.

## 7. Discussion

This study highlights that the CD addresses key challenges in satellite conjunction analysis, particularly probability dilution in probabilistic inference and ambiguity in Neymanian CI procedures, by leveraging its point mass to capture the uncertainty in the data. This distinguishes the CD from other probabilistic approaches, such as UP and RP, which fail to maintain the confidence feature in satellite conjunction analysis and Stein’s length problem. Additionally, the CD provides a direct interpretation of epistemic confidence for hypothesis testing.

The CD can provide reliable decision-making on whether an avoidance maneuver is necessary or not by overcoming probability dilution, but the current work is based on the typical model assumptions based on Gaussian distribution and fast encounter [2,13,25]. The distribution of possible positions of the two satellites at the time of closest encounter is widely assumed to have a multivariate normal distribution with a given mean and covariance matrix [26]. However, Sánchez et al. [39] pointed out that the Gaussian assumption may not always hold and improved the classification approach of Sánchez and Vasile [40]. As an anonymous referee pointed out, the decision to execute an avoidance maneuver may not be strictly binary and there could be various other considerations and metrics. In particular, a broader framework for space traffic coordination highlights the need for incorporating multiple sources of space situational awareness data, maneuverability characteristics, and risk abatement strategies [41].

The CD depends on the underlying distributional assumptions. CD for different assumptions can be derived using pivotal quantities, ancillary statistics [32], or approximation methods [42]. In settings where the true location trajectory is given as a temporal sequence, a predictive distribution [11] can be adapted to extend CD to account for temporal correlations, better capturing the evolution of positional uncertainty over time. These considerations suggest possible directions for interesting future research to further develop CD-based inference for broader applications in space situational awareness.

## Data Availability

No new data were created or analyzed in this study. Data sharing is not applicable to this article.

## Abbreviations

The following abbreviations are used in this manuscript:

CI	Confidence interval
CD	Confidence distribution
RP	Reference posterior
UP	Posterior under uniform (flat) prior
FD	Fiducial distribution
GFD	Generalized fiducial distribution

## Appendix A. Proof of Theorem 1

**Proof.** Let $D_{U}$ and $D_{L}$ be the upper and lower bounds of $\Omega_{D}$, and let $\theta_{U}$ and $\theta_{L}$ be the upper and lower bounds of Θ. Since the quantile function is continuous, we write $q_{\alpha }\left(\theta_{L}\right)=\lim_{\theta \to \theta_{L}}q_{\alpha }\left(\theta \right)$ and $q_{\alpha }\left(\theta_{U}\right)=\lim_{\theta \to \theta_{U}}q_{\alpha }\left(\theta \right)$. Note here that we allow the bounds to be ±∞.

(⇒) Suppose that there exists 0≤α≤1 such that

$$q_{\alpha }\left(\theta_{L}\right)\neq D_{L}\;\mathrm{or}\;q_{\alpha }\left(\theta_{U}\right)\neq D_{U}.$$

If $q_{\alpha }\left(\theta_{L}\right)\neq D_{L}$, there exists $d^{*}$ such that $D_{L}<d^{*}<q_{\alpha }\left(\theta_{L}\right)$. Since $C(\theta ;d^{*})$ does not have a point mass,

$$C\left(\theta_{L};d^{*}\right)=P_{\theta_{L}}\left(D\geq d^{*}\right)=0.$$

However, by definition of the quantile,

$$C\left(\theta_{L};d^{*}\right)>C\left(\theta_{L};q_{\alpha }\left(\theta_{L}\right)\right)=P_{\theta_{L}}\left(D\geq q_{\alpha }\left(\theta_{L}\right)\right)=\alpha >0.$$

This leads to contradiction. If $q_{\alpha }\left(\theta_{U}\right)\neq D_{U}$, there exists $d^{*}$ such that $q_{\alpha }\left(\theta_{U}\right)<d^{*}<D_{U}$. Similarly,

$$C\left(\theta_{U};d^{*}\right)=P_{\theta_{U}}\left(D\geq d^{*}\right)=1,$$

but we have

$$C\left(\theta_{U};d^{*}\right)<C\left(\theta_{U};q_{\alpha }\left(\theta_{U}\right)\right)=P_{\theta_{U}}\left(D\geq q_{\alpha }\left(\theta_{U}\right)\right)=\alpha <1,$$

which leads to contradiction. Thus, $q_{\alpha }\left(\theta \right)\to \partial \Omega_{D}$ as θ→∂Θ for all α∈(0,1).

(⇐) Let *d* be an arbitrary value in $\Omega_{D}$, then (15) leads to

$$C\left(\theta_{L};d\right)=P_{\theta_{L}}\left(D\geq d\right)\geq P_{\theta_{L}}\left(D\geq q_{\alpha }\left(\theta_{L}\right)\right)=\alpha .$$

Taking α=0 leads to $C(\theta_{L};d)=0$. Similarly we can obtain $C(\theta_{U};d)=1$. Thus, C(θ;d) does not have a point mass for any $d\in \Omega_{D}$. □

## Appendix B. Null Belief Problem in CB

This section demonstrates that CB may not be suitable for directly making a binary decision rule. As shown in Figure 4 of Section 5.3, the consonant feature is not the key to overcoming probability dilution. Section 6.2 demonstrates that it is unnecessary to prevent false confidence for all possible propositions; it is sufficient to address false confidence for the proposition of interest. This section further notes that preventing false confidence for all possible propositions may lead to another side effect, tentatively referred to as *null belief*.

In the Dempster-Shafer framework [34,35], belief about a proposition is represented as an interval bounded by the belief function and the plausibility function. As derived in Section 5.3, a consonant belief function for a proposition *A* is defined as follows [2]:

$$\mathrm{Bel}\left(A;d\right)=1-\underset{\theta \in A^{c}}{\sup}\left\{1-|2C\left(\theta ;d\right)-1|\right\}.$$

Then, the plausibility function is given by

(A1) $$\mathrm{Pl}\left(A;d\right)=1-\mathrm{Bel}\left(A^{c};d\right)=\underset{\theta \in A}{\sup}\{1-|2C\left(\theta ;d\right)-1\left|\right\}.$$

To provide justification for the use of CB, Balch et al. [2] introduced the false confidence theorem; however, we can construct an opposite theorem based on similar reasoning. It is worth noting that the following theorem merely points out the trade-off, as overly reducing Type II error can result in a large Type I error.

**Theorem** **A1** (Null belief theorem). *Consider a CB Bel(·;d) characterized by either a CD or posterior probability. Then, for any true $\theta_{0}\in \Theta$ and any p∈(0,1), there exists an interval I with positive length such that*

(A2) $$\theta_{0}\in I\subset \Theta \;\mathrm{and}\;P_{\theta_{0}}\left\{\mathrm{Bel}\left(I;D\right)=0\right\}\geq p.$$

**Proof.** First, take a small interval near the true $\theta_{0}$. Let $\hat{\theta }\left(d\right)$ be the median of the CD such that $C\left(\hat{\theta }\left(d\right);d\right)=0.5$. Then, for any p∈(0,1), there exists ϵ>0 such that

$$P_{\theta_{0}}\left\{\hat{\theta }\left(D\right)\in \left(\theta_{0}-\epsilon ,\theta_{0}+\epsilon \right)\right\}\leq 1-p.$$

Let $I=(\theta_{0}-\epsilon ,\theta_{0}+\epsilon )$ be an interval that contains the true value $\theta_{0}$ but is such that

$$P_{\theta_{0}}\left\{\mathrm{Bel}\left(I;D\right)=0\right\}=P_{\theta_{0}}\left\{\hat{\theta }\left(D\right)\notin I\right\}\geq p,$$

then the theorem is proved. □

In satellite conjunction analysis, the CB of collision, $\mathrm{Bel}\left(H_{0}\right)=\mathrm{Bel}\left(\left[0,R\right]\right)$, often becomes zero due to the null belief theorem. For instance, when the true value is $\theta_{0}=R$ (collision),

$$P_{\theta_{0}=R}\left\{\mathrm{Bel}(H_{0})=0\right\}=P_{\theta_{0}=R}\left\{C(R;D)\leq 0.5\right\}=0.5.$$

Thus, the belief of collision becomes zero with probability 1/2. From the relationship between the belief function and the plausibility function,

(A3) $$\mathrm{Pl}\left(A;d\right)=1-\mathrm{Bel}\left(A^{c};d\right),$$

the corresponding plausibility function is shown to suffer from a similar issue.

In satellite conjunction analysis, the decision is often considered binary: whether to perform an avoidance maneuver (accept $H_{0}$) or not (reject $H_{0}$). There would be no option for uncertainty or indeterminate decisions. Now, suppose that we construct a binary decision rule based on CB, where $H_{0}$ is rejected if {Bel$\left(H_{0})\leq \alpha \right\}$ for some predetermined α. Then,

$$P_{\theta_{0}=R}\left(\mathrm{Reject}\;H_{0}\right)=P_{\theta_{0}=R}\left\{\mathrm{Bel}(H_{0})\leq \alpha \right\}\geq P_{\theta_{0}=R}\left\{\mathrm{Bel}(H_{0})=0\right\}=0.5.$$

Thus, though $H_{0}$ (collision) is true, the CB rejects $H_{0}$ with probability 0.5. It implies that the CB cannot achieve the significance level under 0.5. Similarly, Bel_*G*_(*H*_0_) cannot achieve the significance level under 0.847. In consequence, using CB to prevent false confidence for all possible propositions may not be appropriate for binary decision problems in satellite conjunction analysis.
